# PFOS Exposure Triggers NRF2-Mediated Senescence in Bone Marrow Mesenchymal Stem Cells to Attenuate Their Chondrogenic Potential

**DOI:** 10.3390/toxics14070575

**Published:** 2026-06-30

**Authors:** Hengxia Zheng, Han Zhou, Huawei Liu, Shan Hua, Yilong Wang

**Affiliations:** The Institute for Biomedical Engineering & Nano Science, School of Medicine, Tongji University, Shanghai 200092, China; zzzzzzhx@tongji.edu.cn (H.Z.); hannahz@tongji.edu.cn (H.Z.); 2332177@tongji.edu.cn (H.L.)

**Keywords:** perfluorooctane sulfonate, Bone marrow mesenchymal stem cell, cellular senescence, Nrf2/HO-1 pathway, GelMA hydrogel, cartilage tissue engineering

## Abstract

The widespread application of per- and polyfluoroalkyl substances (PFASs) has established perfluorooctanesulfonic acid (PFOS), a representative PFAS, as a critical environmental pollutant. Although PFOS exposure causes significant bioaccumulation and potential myelotoxicity, its specific impact on the chondrogenic differentiation of bone marrow mesenchymal stem cells (BMSCs) remains to be elucidated. In this study, we established a murine model of PFOS exposure to isolate primary BMSCs and investigated this issue through in vitro differentiation assays, cellular senescence evaluations, and an in vivo subcutaneous implantation model using gelatin methacryloyl (GelMA) hydrogel scaffolds. Our results demonstrated that PFOS exposure triggered intracellular reactive oxygen species (ROS) accumulation and induced a senescent phenotype in BMSCs, characterized by restricted cellular proliferation and the release of senescence-associated secretory phenotype (SASP) factors, thereby markedly suppressing their chondrogenic capacity. Mechanistically, the inhibition of the Nrf2 signaling pathway by PFOS was identified as the principal driver of this process. Furthermore, both in vitro and in vivo assays confirmed that pharmacological activation using the Nrf2 agonist sulforaphane (SFN) effectively mitigated the senescent phenotype and restored the chondrogenic potential of PFOS-exposed BMSCs. Altogether, these findings elucidate the specific mechanisms of PFOS-induced stem cell toxicity and offer a potential strategy to overcome the resulting limitations in BMSC-based cartilage regeneration.

## 1. Introduction

Cartilage is a highly specialized, avascular, and aneural tissue characterized by slow extracellular matrix turnover and a severely limited capacity for self-repair [1]. Clinically, cartilage regeneration is frequently required for conditions including degenerative joint or intervertebral disc diseases, microtia, nasal defects, and temporomandibular joint (TMJ) disorders, among others. Current clinical strategies for cartilage repair rely primarily on autologous cartilage transplantation or the isolation and in vitro expansion of primary chondrocytes to construct tissue-engineered cartilage scaffolds. However, these approaches are hindered by inherent limitations, such as donor-site morbidity, limited cell availability, and the propensity for chondrocyte dedifferentiation during in vitro expansion, often leading to unsatisfactory long-term regenerative outcomes [2,3]. Consequently, conventional strategies remain largely inadequate for achieving high-quality cartilage tissue regeneration within the complex native cartilage microenvironment. Bone marrow mesenchymal stem cells (BMSCs) are multipotent adult stem cells residing within the bone marrow microenvironment, characterized by robust multilineage differentiation potential. Owing to advantages including minimally invasive isolation, rapid in vitro expansion, and low immunogenicity, BMSCs are recognized as a highly promising cell source and are widely applied in cartilage tissue engineering [4]. Nevertheless, the biological activity of BMSCs is highly susceptible to local microenvironmental fluctuations. Even trace amounts of adverse stimuli can induce aberrant phenotypic alterations and shift their differentiation trajectory, thereby impeding efficient chondrogenesis in engineered cartilage tissues [5,6].

Recently, the bioaccumulation of emerging environmental pollutants, notably per- and polyfluoroalkyl substances (PFASs), has become a major public health concern. Owing to their exceptional hydrophobic, oleophobic, and thermostable properties, PFASs are extensively utilized in consumer products. Because they are highly resistant to environmental degradation and human metabolism, PFASs are frequently termed “forever chemicals” [7,8]. Among them, perfluorooctanesulfonic acid (PFOS) is one of the most representative PFASs. Beyond their widely recognized hepatotoxicity, thyroid toxicity, neurotoxicity, and endocrine-disrupting effects, the impact of PFASs on the skeletal and cartilaginous systems is garnering increasing attention. Multiple studies indicate that PFASs can penetrate tissue barriers and accumulate within the bone marrow microenvironment [9,10], a phenomenon closely correlated with osteoarthritis and altered bone mineral density [11,12,13]. Furthermore, recent findings reveal that PFASs significantly influence the fate of embryonic stem cells [14], the osteogenic and adipogenic potential of mesenchymal stem cells [15], and the integrity of pluripotent stem cells [16]. These observations prompted us to consider whether PFOS affects the phenotype of BMSCs. Consequently, it is imperative to elucidate whether PFOS exposure compromises the chondrogenic differentiation potential of BMSCs, thereby affecting the quality of regenerated cartilage, and to identify the underlying molecular mechanisms.

To address this, we isolated primary BMSCs from murine bone marrow and demonstrated that exposure to PFOS attenuates their chondrogenic differentiation capacity. Given the detrimental impact of cellular senescence on chondrogenesis and the observed proliferation inhibition following PFOS exposure, we investigated the in vitro effects of PFOS on BMSC senescence, focusing on the regulatory role of the Nrf2 signaling pathway. Furthermore, as most existing PFOS research is restricted to epidemiological observations and in vitro assays, we fabricated BMSC-laden hydrogel scaffolds to evaluate the in vivo impact of PFOS exposure on chondrogenesis. Collectively, this study provides fundamental insights into PFOS-induced stem cell toxicity and its implications for BMSC-based cartilage regeneration.

## 2. Materials and Methods

### 2.1. Establishment of the PFOS Exposure Murine Model

Six-week-old male C57BL/6 mice (Biotech-Yuekang Life Technology (Shanghai) Co., Ltd., Shanghai, China) were housed in a specific pathogen-free (SPF) environment at the animal facility of Tongji University School of Medicine. The facility was maintained at an ambient temperature of 21–22 °C under a 12-h light/dark cycle, with ad libitum access to standard chow and water. Mice were randomly allocated to either the PFOS (Sigma-Aldrich, Darmstadt, Germany) exposure group (intraperitoneal [i.p.] injection of 10 mg/kg PFOS dissolved in PBS containing 2% Tween-80, once daily [q.d.] for 28 days) or the control group (i.p. injection of an equivalent volume of PBS containing 2% Tween-80) [17]. The physiological condition and behavioral patterns of the mice were monitored daily. All animal procedures were strictly conducted in compliance with the guidelines of the Institutional Animal Care and Use Committee (IACUC). The experimental protocols were approved by the Tongji University Animal Ethics Committee (Approval No. TJAA09726101). The animals were provided by Biotech-Yuekang Life Technology (Shanghai) Co., Ltd. and housed in the SPF animal facility of Tongji University School of Medicine. Humane endpoints included persistent pain, severe localized inflammation, or rapid weight loss exceeding 20%.

### 2.2. Isolation and Culture of BMSCs

Mice were anesthetized using 3–4% isoflurane and maintained with 1.5–2.0% isoflurane via a facemask, followed by euthanasia via cervical dislocation. Bilateral humeri and tibiae were excised under aseptic conditions, and adherent soft tissues were removed. The metaphyseal ends were transected to expose the marrow cavity, and the bone marrow was gently flushed using a 1-mL syringe filled with complete culture medium. Following centrifugation, the cell suspension was plated. After 48 h, non-adherent hematopoietic cells and debris were removed by washing with sterile PBS. The remaining adherent cells, designated as BMSCs, were cultured in complete α-MEM (Gibco, Grand Island, NY, USA) and passaged upon reaching 80–90% confluence. For directed differentiation, BMSCs were cultured in specific chondrogenic, osteogenic, or adipogenic induction medium (OriCell, Shanghai, China).

### 2.3. Flow Cytometry

BMSCs were harvested using 0.25% trypsin (Gibco, USA) and washed with PBS. For cell cycle analysis, cells were fixed in 70% ethanol at 4 °C for 12 h, then stained with propidium iodide (PI) and RNase A (Beyotime, Cat #C1052, Shanghai, China) for 30 min in the dark. To assess BMSC surface markers, unfixed cells were incubated with fluorochrome-conjugated antibodies against CD29 (Cat #102205, 0.05 μg/mL), CD44 (Cat #156007, 0.025 μg/mL), CD90 (Cat #166405, 0.0015 μg/mL), CD45 (Cat #157608, 0.01 μg/mL), CD11b (Cat #101211, 0.01 μg/mL), and CD34 (Cat #128612, 0.05 μg/mL) (BioLegend, San Diego, CA, USA) for 30 min in the dark. To evaluate intracellular reactive oxygen species (ROS) levels, the harvested cell suspensions were incubated with 10 μM DCFH-DA probe (Beyotime, Cat #S0034S, China) at 37 °C for 20 min in the dark. Subsequently, the cells were washed with PBS and immediately analyzed via flow cytometry. Data were acquired using a flow cytometer (LSRFortessa, BD Biosciences, San Jose, CA, USA), collecting a minimum of 10,000 events per sample, and analyzed utilizing FlowJo software (v10.8.1, BD Biosciences, USA).

### 2.4. Histological Staining

Cell coverslips or tissue samples were fixed in 4% paraformaldehyde, embedded in paraffin, and sectioned. Following deparaffinization and antigen retrieval, sections were incubated with 3% H_2_O_2_ for 25 min at room temperature to quench endogenous peroxidase activity. After blocking with bovine serum albumin (BSA) (Beyotime, China), sections were incubated overnight at 4 °C with a primary antibody against type II collagen (Bioss, Beijing, China). Subsequently, a biotin-conjugated secondary antibody (Beyotime, China) was applied for 30 min at room temperature. Immunoreactivity was visualized using 3,3′-diaminobenzidine (DAB), and nuclei were counterstained with hematoxylin. Sections were then dehydrated through a graded ethanol series, cleared in xylene, and mounted. Additionally, hematoxylin and eosin (H&E), Alcian blue, and Safranin O staining (Servicebio, Wuhan, China) was performed to evaluate cartilaginous matrix deposition.

### 2.5. CCK-8 Assay

BMSCs were seeded into 96-well plates at a density of 4000 cells/well in 100 μL of culture medium. At 24, 48, and 72 h, 10 μL of CCK-8 reagent (Beyotime, China) was added to each well, followed by a 1-h incubation at 37 °C. The absorbance was measured at 450 nm using a microplate reader (Agilent, Santa Clara, CA, USA). To determine the half-maximal inhibitory concentration (IC50), BMSCs were treated with various concentrations of PFOS for 24, 48, or 72 h. Cell viability was calculated based on relative absorbance, and the IC50 value was determined using GraphPad Prism software version 10.1.0.

### 2.6. Western Blotting

Total protein from cells or tissues was extracted using a total protein extraction kit (Boxbio Science & Technology Co., Ltd., Beijing, China), and concentrations were quantified via BCA assay (Beyotime, China) to ensure equal loading. Samples probing for Col2a1, ACAN (HUABIO, Hangzhou, China), and SOX9 (Bioss, China) were denatured at 37 °C for 20 min; all other samples were denatured at 100 °C for 10 min. Equal amounts of protein (15 μg per lane) were separated by SDS-PAGE (Epizyme, Shanghai, China) and transferred onto nitrocellulose membranes (Millipore, Billerica, MA, USA). Membranes were blocked with a universal rapid blocking buffer (Epizyme, China) for 20 min at room temperature and incubated with primary antibodies overnight at 4 °C. Following TBST washes, membranes were incubated with species-specific HRP-conjugated secondary antibodies (Proteintech, Rosemont, IL, USA) for 2 h at room temperature. Immunoreactive bands were visualized using an enhanced chemiluminescence (ECL) reagent (New Cell & Molecular Biotech, Suzhou, China) and captured on a chemiluminescence imaging system (Tanon, Shanghai, China). Densitometric analysis was performed using ImageJ software version 1.8.0 (National Institutes of Health, Bethesda, MD, USA). Primary antibodies included Col2a1 (Bioss, #bs-10589R, 1:1000, China), ACAN (Bioss, #bs-41346R, 1:1000, China), SOX9 (Bioss, #bsm-63031R, 1:2000, China), p16 (Abcam, #ab211542, 1:2000, Waltham, MA, USA), p21 (Abcam, #ab109199, 1:1000, USA), Nrf2 (Cell Signaling Technology, #12721, 1:1000, Danvers, MA, USA), HO-1 (Abcam, #ab68477, 1:5000, USA), and NQO1 (Beyotime, #AF7614, 1:1000, China). All assays were conducted with three independent biological replicates.

### 2.7. Quantitative Real-Time PCR (qRT-PCR)

Total RNA was extracted from BMSCs at approximately 90% confluence using TRIzol reagent (Invitrogen, Carlsbad, CA, USA). Complementary DNA (cDNA) was synthesized using the PrimeScript RT Master Mix kit (Takara, Kusatsu, Japan). qPCR was performed on a fluorescence quantitative PCR system (Thermo Fisher Scientific, Waltham, MA, USA) using the BeyoFast SYBR Green qPCR Mix (Beyotime, China). GAPDH was utilized as the endogenous reference gene for normalization. Primer (Sangon, Shanghai, China) sequences are detailed in Table S1. Relative mRNA expression levels were calculated using the 2^−ΔΔCt^ method.

### 2.8. Immunofluorescence Staining

BMSCs seeded on glass coverslips were fixed with 4% paraformaldehyde, permeabilized with 0.2% Triton X-100, and blocked with 5% goat serum. Cells were incubated with primary antibodies overnight at 4 °C. Filamentous actin (F-actin) was labeled with FITC-conjugated phalloidin (Beyotime, China). Subsequently, cells were incubated with corresponding fluorophore-conjugated secondary antibodies for 1 h at room temperature in the dark, and nuclei were counterstained with Hoechst 33342 (Beyotime, China). Images were acquired using a laser confocal microscope (Leica Microsystems, Wetzlar, Germany). For tissue immunofluorescence, deparaffinized and rehydrated sections underwent antigen retrieval in 0.01 M sodium citrate buffer (pH 6.0), were blocked with 5% goat serum containing 0.3% Triton X-100, and were sequentially incubated with primary antibodies, secondary antibodies, and DAPI before fluorescence microscopy evaluation. Both mean fluorescence intensity and Nrf2 nuclear translocation were quantified using ImageJ software across at least three random fields per group. The translocation ratio was calculated as the percentage of cells displaying predominant nuclear Nrf2 fluorescence relative to the total cell count. Data were statistically analyzed and graphed using GraphPad Prism.

### 2.9. Senescence-Associated β-Galactosidase (SA-β-gal) Staining

Adherent BMSCs were fixed with a cell fixation buffer for 20 min at room temperature and subsequently washed with PBS. The cells were then treated with an SA-β-gal staining working solution (Beyotime, China), prepared according to the manufacturer’s instructions, and incubated at 37 °C for 12 h. Stained cells were observed and imaged under a light microscope.

### 2.10. Enzyme-Linked Immunosorbent Assay (ELISA)

Cell culture supernatants were collected to quantify senescence-associated secretory phenotype (SASP) markers, including IL-1β (Elabscience, Wuhan, China), IL-6 (Jianglai, Shanghai, China), IL-8 (Elabscience, China), and MMP3 (Abcam, USA). ELISA was performed by sequentially incubating samples with capture and detection antibodies according to the manufacturer’s instructions. Absorbance values were measured using a multimode microplate reader, and the concentrations were calculated against standard curves generated via GraphPad Prism software.

### 2.11. Construction of BMSC-Laden Hydrogel Cartilage Scaffolds

GelMA (10% *w*/*v*) (EFL, Suzhou, China) was dissolved in sterile PBS containing 0.2% (*w*/*v*) lithium phenyl-2,4,6-trimethylbenzoylphosphinate (LAP) at 50 °C. BMSCs were harvested and resuspended in the GelMA solution at 37 °C at a density of 1 × 10^7^ cells/mL. The BMSC-laden bioink was cast into cylindrical molds and crosslinked under 405 nm light for 15 s. The fabricated hydrogel scaffolds were temporarily maintained in serum-free α-MEM medium prior to in vivo implantation.

### 2.12. In Vivo Animal Experiments

Six-week-old C57BL/6 mice were randomly allocated to experimental groups (*n* = 6 per group). To minimize surgical bias, all procedures were performed by a single investigator. Mice were anesthetized using 2% isoflurane. Following dorsal depilation and disinfection with iodophor, a 1-cm skin incision was made, and a subcutaneous pocket was bluntly dissected for the implantation of the cylindrical hydrogel scaffold. The incision was closed with 5-0 nylon sutures and treated topically with erythromycin ointment. One-month post-implantation, mice were euthanized via cervical dislocation following deep anesthesia, and the scaffolds were harvested for histological evaluation.

### 2.13. Statistics

Data are expressed as the mean ± standard deviation. Statistical analyses were conducted using GraphPad Prism 10.6.0 software. The normality of data distribution and homogeneity of variance were verified prior to further testing. For comparisons between two independent groups, Student’s *t*-test was used. For multiple groups, statistical significance was assessed by one-way ANOVA followed by Tukey’s post hoc test for pairwise comparisons. A *p*-value < 0.05 was considered statistically significant, and the levels of significance were denoted as follows: * *p* < 0.05, ** *p* < 0.01, *** *p* < 0.001, and ns (not significant). All experiments were independently repeated a minimum of three times per group. All *n* values, including animal-derived samples, represent independent biological replicates. Technical replicates were averaged to yield a single data point per experiment.

## 3. Results

### 3.1. Isolation and Characterization of C57 Mouse BMSCs and Establishment of the PFOS Exposure Model

**Figure 1:**
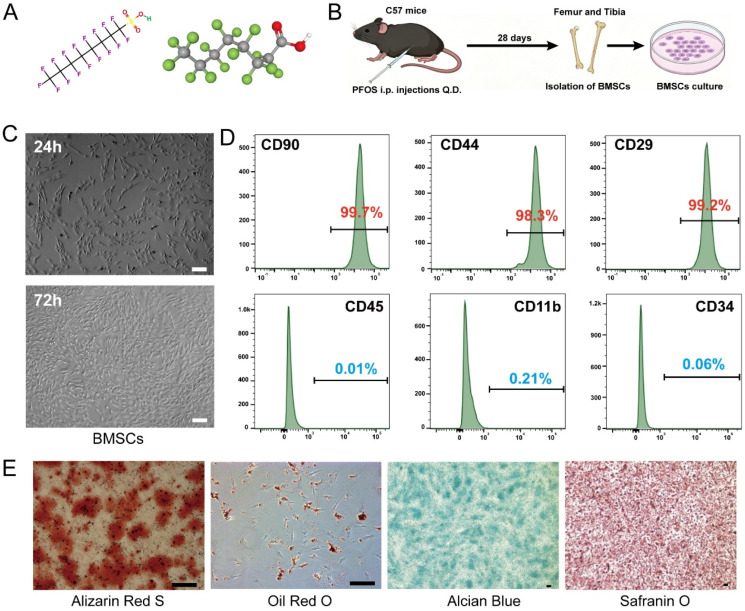
(**A**) 2D and 3D chemical structures of PFOS. (**B**) Schematic illustration of the in vivo PFOS exposure murine model and in vitro BMSC isolation. (**C**) Representative inverted phase-contrast microscopy image of P3 BMSCs. Scale bar: 100 μm. (**D**) Flow cytometric analysis of typical surface marker expression in P3 BMSCs. (**E**) Assessment of the multilineage differentiation potential of BMSCs. Scale bar: 100 μm.

The chemical structure of PFOS is presented in Figure 1A. Following continuous PFOS exposure for 28 days, bone marrow cells from C57BL/6 mice were harvested for adherent culture and expanded to passage 3 (P3) for subsequent experiments (Figure 1B). Phase-contrast microscopy revealed a small number of adherent cells with primarily spindle or polygonal morphologies after 24 h. By 72 h, the cells exhibited significant proliferation and formed the typical fibroblast-like, whorl-shaped colonies characteristic of BMSCs (Figure 1C). Flow cytometric analysis confirmed their BMSC identity, showing robust expression of mesenchymal markers (CD90, CD44, and CD29 > 98%) and minimal expression of hematopoietic markers (<0.5%) (Figure 1D). Furthermore, multilineage differentiation assays confirmed that control BMSCs successfully differentiated into osteogenic, adipogenic, and chondrogenic lineages (Figure 1E). Collectively, these findings confirm the successful isolation of functional murine BMSCs for subsequent exposure modeling.

### 3.2. In Vivo PFOS Exposure Significantly Inhibits the Chondrogenic Differentiation Potential of Ex Vivo BMSCs

**Figure 2:**
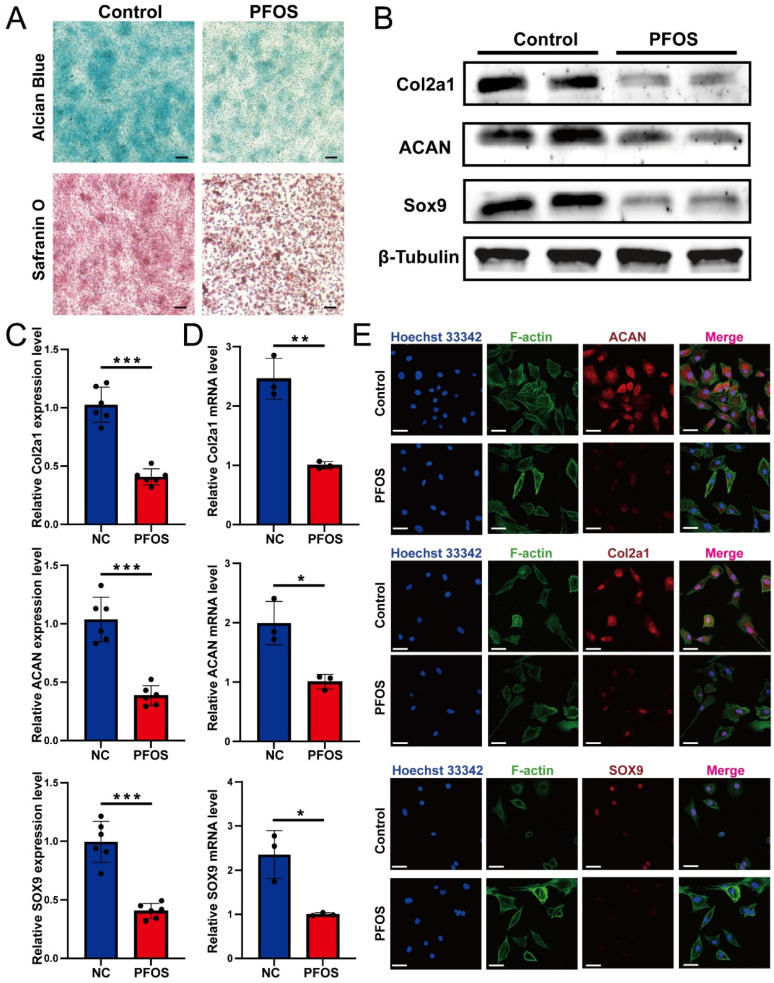
(**A**) Representative Alcian Blue and Safranin O staining of BMSCs from control and PFOS-exposed C57 mice following chondrogenic induction. Scale bar: 100 μm. (**B**) Western blot analysis of cartilage-specific markers (Col2a1, ACAN, and SOX9) in BMSCs from control and PFOS-exposed mice following chondrogenic induction. (**C**) Semi-quantitative densitometric analysis of the Western blot results. *n* = 6. (**D**) qRT-PCR analysis of Col2a1, ACAN, and SOX9 mRNA expression levels in the two groups. *n* = 3. (**E**) Immunofluorescence evaluation of the relative expression of chondrogenic markers in BMSCs. Magnification: 63×; Scale bar: 25 μm. * *p* < 0.05, ** *p* < 0.01, *** *p* < 0.001.

To determine whether PFOS exposure compromises BMSC chondrogenesis, ex vivo BMSCs from control and PFOS-exposed mice were subjected to chondrogenic induction. Compared to the pronounced glycosaminoglycan and cartilage matrix deposition in control cells, the PFOS-exposed group exhibited attenuated matrix formation (Figure 2A). Western blot analysis demonstrated that the protein expression levels of the cartilage-specific markers Col2a1, ACAN, and SOX9 were significantly decreased in the PFOS-exposed group relative to the control (Figure 2B,C). Consistently, qRT-PCR and immunofluorescence analyses confirmed downregulation of Col2a1, ACAN, and SOX9 at both mRNA and protein levels (Figure 2D,E and Figure S1). Taken together, these findings indicate that in vivo PFOS exposure impairs the chondrogenic differentiation capacity of murine BMSCs.

### 3.3. In Vivo PFOS Exposure Induces Cellular Senescence in Ex Vivo BMSCs

**Figure 3:**
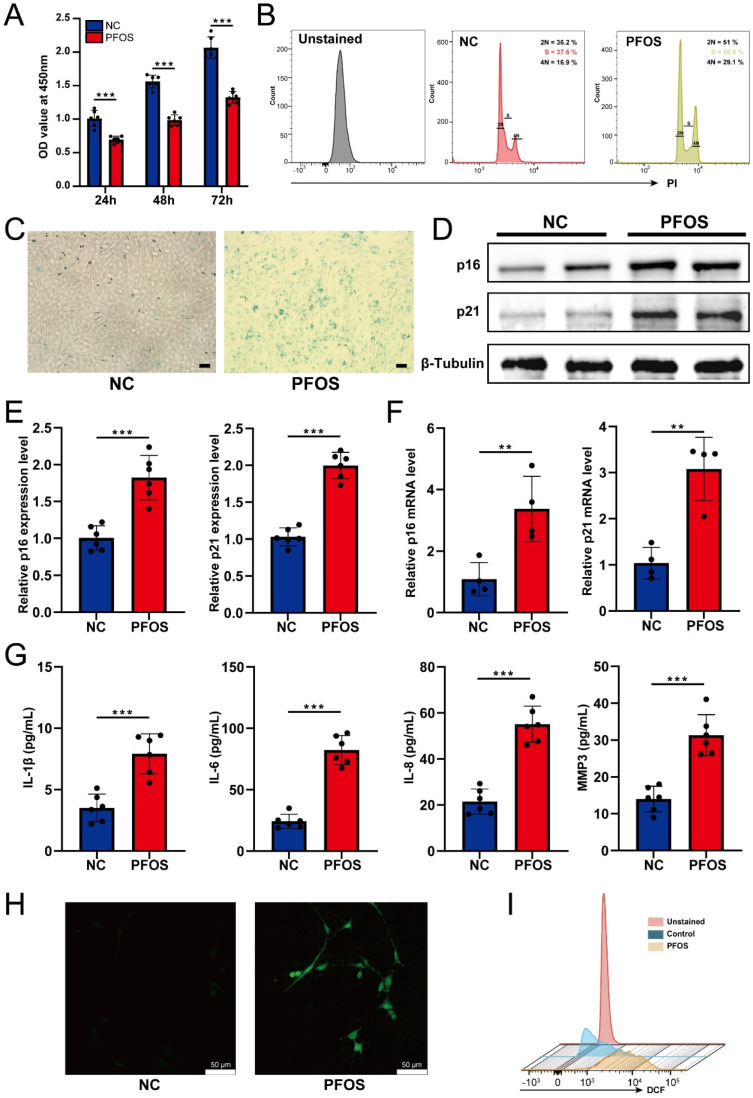
(**A**) CCK-8 assay comparing the proliferation of P3 BMSCs between the two groups. *n* = 6. (**B**) Flow cytometric analysis of cell cycle distribution in the two groups. (**C**) SA-β-gal staining comparing cellular senescence between the two groups. Scale bar: 100 μm. (**D**) Western blot analysis of the senescence-associated markers p16 and p21. (**E**) Semi-quantitative densitometric analysis of the Western blot results. *n* = 6. (**F**) qRT-PCR analysis of p16 and p21 mRNA expression levels in the two groups. *n* = 4. (**G**) ELISA quantification of SASP markers, including IL-1β, IL-6, IL-8, and MMP3, in the cell culture supernatants of both groups. *n* = 6. (**H**) Intracellular ROS levels in BMSCs were observed via laser confocal microscopy. Magnification: 40×; Scale bar: 50 μm. (**I**) Flow cytometric analysis of intracellular ROS levels in BMSCs. ** *p* < 0.01, *** *p* < 0.001.

Given the association between cellular senescence and diminished differentiation capacity, we investigated the effects of PFOS exposure on BMSC proliferation and senescence. PFOS exposure significantly suppressed BMSC viability (Figure 3A) and induced cell cycle arrest at the G0/G1 and G2/M phases (Figure 3B). PFOS triggered a senescent phenotype, characterized by increased senescence-associated β-galactosidase (SA-β-gal) activity (Figure 3C) and upregulation of senescence-associated markers (p16 and p21) at both protein and transcript levels (Figure 3D–F). Additionally, ELISA demonstrated that the concentrations of senescence-associated secretory phenotype (SASP) factors, including IL-1β, IL-6, IL-8, and MMP3, were significantly elevated in the culture supernatants of PFOS-exposed BMSCs (Figure 3G). Given that oxidative stress drives cellular aging, intracellular ROS accumulation was evaluated. Compared to the baseline control, PFOS-exposed BMSCs exhibited a marked surge in oxidative stress, as evidenced by a pronounced increase in green fluorescence (Figure 3H) and a distinct rightward shift in flow cytometric intensity (Figure 3I). Together, these data indicate that PFOS exposure impairs BMSC proliferation by provoking intracellular oxidative stress and triggering cellular senescence.

### 3.4. In Vitro PFOS Treatment Dose-Dependently Suppresses the Nrf2/HO-1 Signaling Pathway in BMSCs

**Figure 4:**
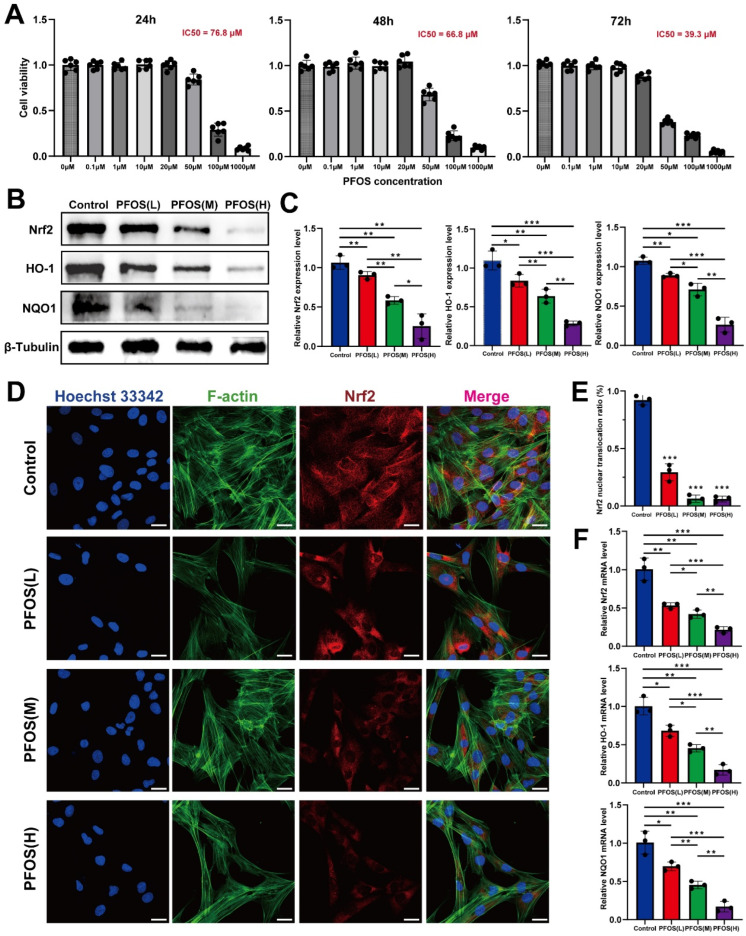
(**A**) Determination of the IC50 of PFOS on BMSCs in vitro using a CCK-8 assay. *n* = 6. (**B**) Western blot analysis evaluating the effect of varying PFOS concentrations on the expression of Nrf2 signaling pathway markers in BMSCs. (**C**) Semi-quantitative densitometric analysis of the Western blot results. *n* = 3. (**D**) Immunofluorescence evaluation of Nrf2 expression and nuclear translocation in BMSCs following PFOS treatment. Magnification: 63×; Scale bar: 25 μm. (**E**) Quantitative comparative analysis of the Nrf2 nuclear translocation rate in BMSCs across groups. *n* = 3. (**F**) The mRNA expression levels of Nrf2, HO-1, and NQO1 were detected by qRT-PCR. *n* = 3. * *p* < 0.05, ** *p* < 0.01, *** *p* < 0.001.

To elucidate the molecular mechanisms underlying PFOS-induced BMSC senescence and impaired chondrogenesis, we transitioned to an in vitro experimental paradigm using direct PFOS treatment on BMSCs. PFOS induced a time- and dose-dependent decline in BMSC viability, with IC50 values of 76.8 μM, 66.8 μM, and 39.3 μM at 24, 48, and 72 h, respectively (Figure 4A). Mechanistically, PFOS exposure suppressed the Nrf2/HO-1 antioxidant signaling cascade. Both protein and mRNA levels of Nrf2 and its downstream effectors (HO-1 and NQO1) were dose-dependently downregulated (Figure 4B,C,F). In addition, immunofluorescence showed that PFOS treatment diminished overall Nrf2 expression (Figure S2) and blocked its nuclear translocation (Figure 4D,E).

### 3.5. Activation of Nrf2 Significantly Reverses PFOS-Induced BMSC Senescence and Restores Chondrogenic Capacity

**Figure 5:**
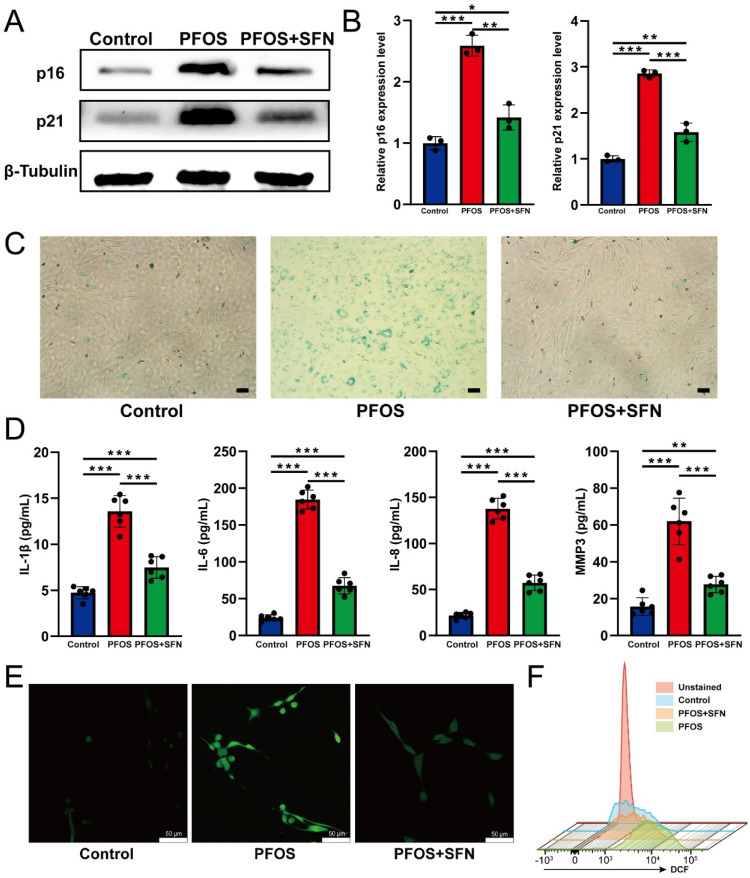
(**A**) Western blot analysis demonstrating the reversal of PFOS-induced p16 and p21 upregulation in BMSCs by SFN treatment. (**B**) Semi-quantitative densitometric analysis of the Western blot results. *n* = 3. (**C**) SA-β-gal staining demonstrating the restorative effect of SFN on PFOS-induced BMSC senescence. Scale bar: 100 μm. (**D**) ELISA demonstrating the reversal of the PFOS-induced increase in BMSC SASP secretion by SFN treatment. *n* = 6. (**E**) Intracellular ROS levels in BMSCs were directly observed via laser confocal microscopy. Magnification: 40×; Scale bar: 50 μm. (**F**) Flow cytometric analysis of intracellular ROS levels in BMSCs. * *p* < 0.05, ** *p* < 0.01, *** *p* < 0.001.

To verify the functional role of the Nrf2 pathway in PFOS-induced senescence, BMSCs subjected to in vitro PFOS exposure were co-treated with the Nrf2 activator SFN. Western blot analysis showed that the elevated expression of p16 and p21 in the PFOS group was significantly attenuated following SFN treatment (Figure 5A,B). Consistent with this, SA-β-gal staining demonstrated a substantial decrease in the number of SA-β-gal-positive cells in the PFOS + SFN group compared to the PFOS-alone group, indicating that SFN mitigates the senescent phenotype (Figure 5C). Furthermore, ELISA revealed that SFN treatment significantly reduced the secretion of SASP factors (IL-1β, IL-6, IL-8, and MMP3) in the culture supernatants (Figure 5D). These results confirm that Nrf2 activation effectively counteracts PFOS-induced BMSC senescence. Importantly, SFN intervention effectively mitigated the PFOS-induced intracellular ROS accumulation, as evidenced by a marked reduction in green fluorescence (Figure 5E) and a restorative leftward shift in the flow cytometric profile (Figure 5F).

**Figure 6:**
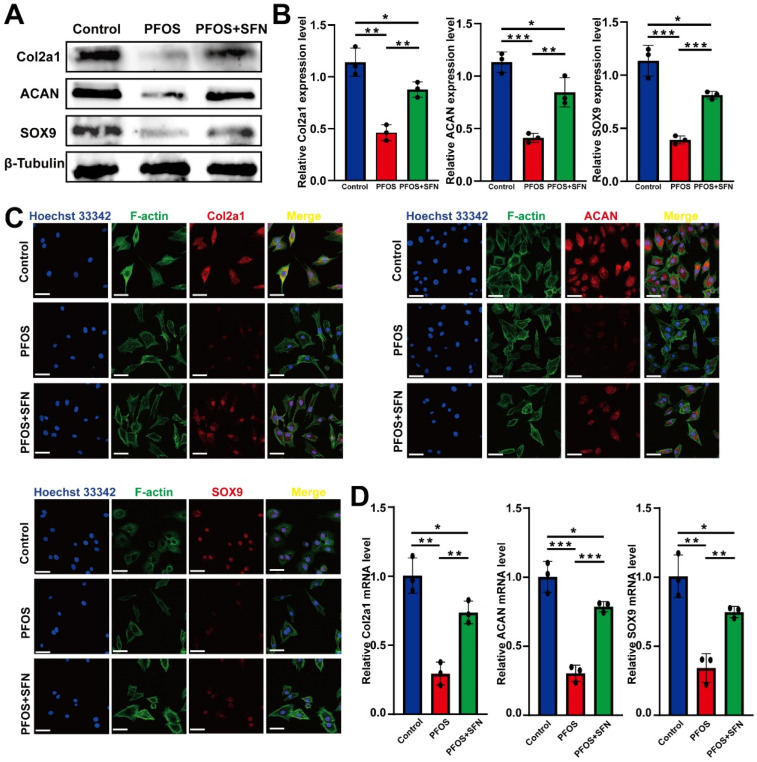
(**A**) Western blot analysis of cartilage-specific marker expression in distinct groups of BMSCs following chondrogenic induction. (**B**) Semi-quantitative densitometric analysis of the Western blot results. *n* = 3. (**C**) Immunofluorescence evaluation of cartilage-specific marker expression in distinct groups of BMSCs following chondrogenic induction. Magnification: 63×; Scale bar: 25 μm. (**D**) qRT-PCR analysis of the mRNA expression levels of cartilage-specific markers in distinct groups of BMSCs following chondrogenic induction. *n* = 3. * *p* < 0.05, ** *p* < 0.01, *** *p* < 0.001.

Furthermore, SFN rescued the chondrogenic capacity of these cells. Following chondrogenic induction, Western blot analysis demonstrated that the PFOS-induced reduction in Col2a1, ACAN, and SOX9 protein expression was markedly reversed by SFN intervention (Figure 6A,B). Immunofluorescence staining similarly revealed that the diminished signals for Col2a1, ACAN, and SOX9 in the PFOS group were substantially enhanced following SFN treatment (Figure 6C and Figure S3). Moreover, qRT-PCR verified that SFN significantly upregulated the mRNA expression of these cartilage markers in PFOS-treated BMSCs (Figure 6D). In summary, pharmacological activation of Nrf2 alleviates PFOS-induced cellular senescence and ameliorates the compromised chondrogenic differentiation capacity of BMSCs.

### 3.6. SFN Rescues Cartilage Regeneration Deficits in Hydrogel Scaffolds Laden with PFOS-Exposed BMSCs

**Figure 7:**
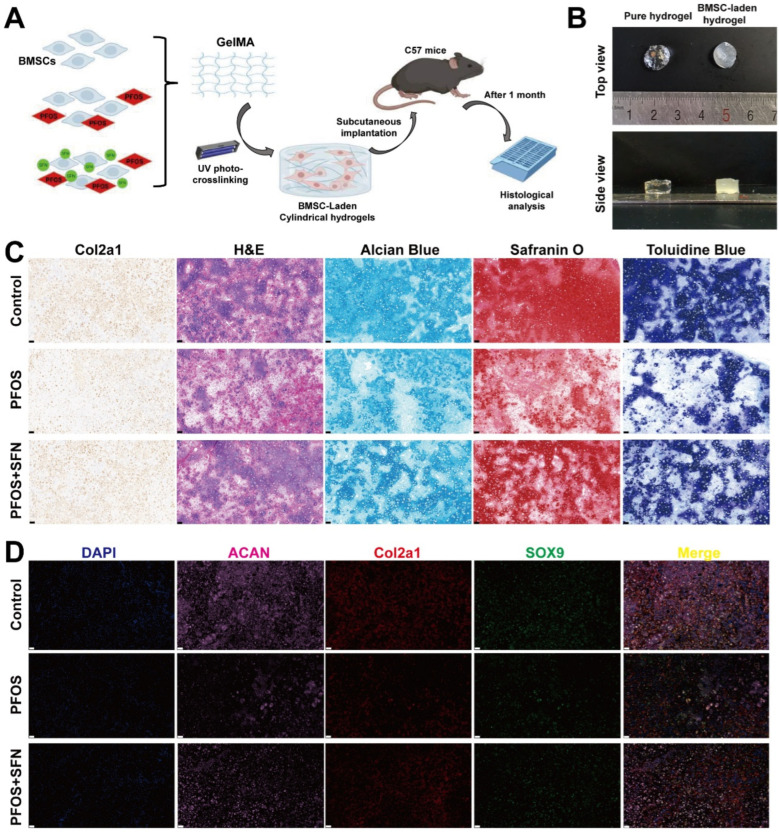
(**A**) Schematic illustration of BMSC-laden hydrogel fabrication and the in vivo experimental design. (**B**) Macroscopic appearance of the cylindrical hydrogel scaffolds. (**C**) In vivo multiplex histological staining of cartilage within the hydrogel scaffolds from different treatment groups. Magnification: 10×; Scale bar: 100 μm. (**D**) In vivo immunofluorescence staining of cartilage-specific markers within the hydrogel scaffolds from different treatment groups. Magnification: 10×; Scale bar: 100 μm.

To evaluate the in vivo relevance of these findings, BMSCs pre-treated in vitro with PFOS and SFN were encapsulated within GelMA hydrogels and subcutaneously implanted into C57BL/6 mice according to the experimental scheme (Figure 7A). The macroscopic appearance of the hydrogel scaffolds is shown in Figure 7B. Histological evaluation via H&E, Alcian Blue, Safranin O, and Toluidine Blue staining showed that while PFOS exposure hindered in vivo cartilage matrix deposition, SFN co-treatment partially restored glycosaminoglycan accumulation (Figure 7C). Tissue immunofluorescence further revealed that the PFOS-induced loss of Col2a1, ACAN, and SOX9 expression within the scaffolds was also partially rescued by SFN intervention (Figure 7D and Figure S4). These in vivo results demonstrate that PFOS exposure impairs BMSC-mediated cartilage regeneration within hydrogel scaffolds, and that Nrf2 activation can ameliorate these regenerative deficits.

## 4. Discussion

The success of cartilage tissue engineering utilizing BMSCs is highly contingent upon cell survival and directed differentiation capacity. However, BMSCs are susceptible to physicochemical perturbations within their local microenvironment [5,6]. Recently, the widespread bioaccumulation of persistent organic pollutants, notably PFASs, in human tissues has emerged as a critical public health concern. Epidemiological and toxicological evidence indicates that PFOS can penetrate the endosteal barrier and accumulate within the bone marrow cavity, an exposure that is positively correlated with decreased bone mineral density and an increased risk of osteoarthritis. This study systematically extends the toxicological evaluation of PFOS, a representative PFAS, into the realm of BMSC-based cartilage regeneration. We demonstrated that PFOS exposure induces BMSC senescence, thereby suppressing their chondrogenic differentiation potential and leading to markedly inefficient in vivo cartilage regeneration within 3D GelMA hydrogel scaffolds. Mechanistically, PFOS inhibits the Nrf2/HO-1 pathway, triggering cellular senescence and the subsequent release of SASP factors, which consequently suppresses the expression of the core chondrogenic transcription factor, SOX9. Furthermore, the application of the specific Nrf2 agonist SFN effectively reversed this pathological process, offering a preemptive intervention strategy for cell-based therapies in the context of environmental pollutant exposure.

To investigate the cytological basis of PFOS-mediated cartilage regeneration failure, we observed that PFOS induced cell cycle arrest and restricted cellular proliferation. Furthermore, this exposure provoked a senescent phenotype characterized by the upregulation of p16 and p21, alongside increased SA-β-gal activity. Stem cell senescence entails not only a decline in self-renewal and multilineage differentiation capacities but also the active secretion of SASP components, including pro-inflammatory cytokines (e.g., IL-1β, IL-6) and matrix-degrading enzymes (e.g., MMP3) [18,19]. Previous investigations into osteoarthritis pathogenesis have established that SASP factors such as IL-6 and MMP3 are central mediators of cartilage matrix degradation. Moreover, they trigger sustained inflammatory cascades via paracrine signaling, thereby inducing secondary senescence and apoptosis in adjacent healthy cells [20,21]. Consistent with these findings, the robust SASP secretion in the PFOS-exposed group significantly impeded Col2a1 assembly, incapacitating the BMSCs from maintaining normal cartilage matrix homeostasis. This finding elucidates why BMSCs originating from a PFOS-exposed environment continue to exhibit conspicuous cartilage regeneration deficits in vivo, even following isolation from the toxicant and subsequent implantation.

To elucidate the molecular mechanisms through which PFOS triggers stem cell senescence and obstructs chondrogenesis, we focused on Nrf2, a master transcriptional regulator of cellular redox homeostasis. Environmental toxicology studies have demonstrated that endocrine-disrupting chemicals, such as PFOS, can incite reactive oxygen species (ROS) accumulation at the mitochondrial respiratory chain, triggering severe DNA damage and lipid peroxidation [21,22,23]. Under physiological conditions, Nrf2 undergoes nuclear translocation to upregulate antioxidant enzymes, notably HO-1, to scavenge ROS and shield cells from toxic insults [24,25]. Our findings reveal that PFOS exposure profoundly suppressed Nrf2 activation and the subsequent expression of its downstream cytoprotective proteins. Furthermore, emerging evidence indicates that activated, nuclear-localized Nrf2 specifically binds to the antioxidant response element (ARE) within the SOX9 promoter region, directly driving the transcriptional activation of SOX9 [26]. SOX9 serves as a key initiating factor directing BMSC commitment to the chondrogenic lineage, directly transactivating the expression of core cartilage matrix genes, such as Col2a1 and ACAN [27,28]. Consequently, the suppression of the Nrf2 pathway by PFOS likely exerts a dual inhibitory effect on BMSCs, leading to the severe attenuation of their chondrogenic differentiation.

Prompted by this strong correlation, we implemented a pharmacological intervention using SFN, a highly specific natural agonist of Nrf2. SFN covalently modifies Keap1, thereby abrogating the ubiquitination and degradation of Nrf2 and facilitating its robust nuclear translocation [29,30]. Our in vitro results demonstrated that SFN pre-treatment significantly downregulated the expression of p16 and p21, and decreased SASP release. Crucially, it reactivated the suppressed SOX9 transcription factor. To validate these in vitro findings within a physiologically relevant microenvironment, we employed GelMA, a 3D biomimetic hydrogel extensively utilized in tissue engineering, for our in vivo assays [31,32]. Subcutaneous implantation experiments revealed that GelMA scaffolds laden with PFOS-exposed BMSCs exhibited markedly reduced cartilage-like matrix deposition. In contrast, PFOS-exposed cells pretreated with SFN deposited a substantially denser network of glycosaminoglycans and cartilaginous matrix within the scaffolds. These findings provide a foundational rationale for pharmacological rescue strategies when considering the clinical application of autologous BMSCs harvested from patients with a history of PFOS exposure.

Although this study provides robust insights into PFOS-induced BMSC toxicity, certain methodological considerations warrant acknowledgment. First, consistent with established methodologies in environmental toxicology, the experimental PFOS doses are higher than typical human exposures. While chronic systemic exposure involves complex systemic toxicokinetics and bone marrow microenvironmental factors compared to direct in vitro acute cellular exposure, such standard regimens are commonly employed to model long-term bioaccumulation and evaluate cumulative mechanisms within a limited experimental timeframe [17]. Second, the incomplete functional rescue by SFN indicates that parallel cascades or mitochondrial alterations likely co-regulate PFOS toxicity [15,33]. Furthermore, because our mechanistic findings rely on pharmacological rather than direct genetic evidence, definitive causality remains to be established in future studies. Finally, although our subcutaneous model is well-suited for evaluating auricular cartilage regeneration, it cannot encompass all types of cartilage repair, such as articular cartilage or meniscus models. This limitation arises because the ectopic subcutaneous platform cannot fully replicate the complex biomechanical loading and specific tissue microenvironments inherent to native joint cavities. Future orthotopic and genetic studies will further validate these findings. In summary, this study demonstrates that suppression of the Nrf2 signaling pathway represents a key molecular mechanism underlying PFOS-induced BMSC senescence and impaired cartilage regeneration, and proposes a viable pharmacological intervention strategy to mitigate this clinical challenge.

## 5. Conclusions

This study demonstrates that PFOS exposure induces cellular senescence in murine BMSCs, a pathological process that is closely associated with the suppression of the Nrf2 signaling pathway. This cascade consequently leads to markedly inefficient chondrogenesis when these compromised BMSCs are utilized in hydrogel scaffolds for cartilage tissue engineering. Notably, targeted pharmacological intervention with the Nrf2 agonist SFN can ameliorate these deleterious outcomes and partially rescue cartilage regeneration potential.

## Data Availability

The original contributions presented in this study are included in the article and Supplementary Material. Further inquiries can be directed to the corresponding authors.

## Supplementary Materials

The following supporting information can be downloaded at: https://www.mdpi.com/article/10.3390/toxics14070575/s1, Figure S1: Relative quantification of ACAN, Col2a1, and Sox9 expression detected by immunoflu-orescence. Figure S2: Relative quantification of Nrf2 expression detected by immunofluorescence. Figure S3: Relative quantification of ACAN, Col2a1, and Sox9 expression detected by immunoflu-orescence. Figure S4: Relative quantification of ACAN, Col2a1, and Sox9 expression in tissues de-tected by immunofluorescence. Table S1: Primer sequences used for qRT-PCR.
