# Supplementary material for: PFOS Exposure Triggers NRF2-Mediated Senescence in Bone Marrow Mesenchymal Stem Cells to Attenuate Their Chondrogenic Potential
Source: Toxics. 2026 Jun 30;14(7):575. doi: 10.3390/toxics14070575 (PMC13418995; doi:10.3390/toxics14070575)
Supplement: Supplementary file 1 [file toxics-14-00575-s001.zip › Table S1.pdf]

Primer	5' to 3'
β-Tubulin-Forward	GGAAATCGTGCACATCCAGG
β-Tubulin-Reverse	GGGGTCGATGCCATGTTCAT
ACAN-Forward	GTGGAGCCGTGTTTCCAAG
ACAN-Reverse	AGATGCTGTTGACTCGAACCT
Col2a1-Forward	CACGTACACTGCCCTGAAGGA
Col2a1-Reverse	CGATAACAGTCTTGCCCCACTT
SOX9-Forward	GTACCCGCACTTGCACAAC
SOX9-Reverse	TCTCGCTCTCGTTCAGAAGTC
p16-Forward	CAGAGCTAAATCCGGCCTCA
p16-Reverse	TCCCTCCCGTGATTGCAAA
p21-Forward	TAAGGACGTCCACCTGTGCC
p21-Reverse	AAAGTTCCACCGTTCTCGGG
Nrf2-Forward	TCTTGGAGTAAGTCGAGAAGTGT
Nrf2-Reverse	GTTGAAACTGAGCGAAAAAGGC
HO-1-Forward	CCCTCTCTCATGCTAGTTCAGC
HO-1-Reverse	GCACAGCGTTTGCTTGACT
HQO1-Forward	AGGATGGGAGGTACTCGAATC
HQO1-Reverse	AGGCGTCCTTCCTTATATGCTA
