# Supplementary figures and images for: PFOS Exposure Triggers NRF2-Mediated Senescence in Bone Marrow Mesenchymal Stem Cells to Attenuate Their Chondrogenic Potential
Source: Toxics. 2026 Jun 30;14(7):575. doi: 10.3390/toxics14070575 (PMC13418995; doi:10.3390/toxics14070575)

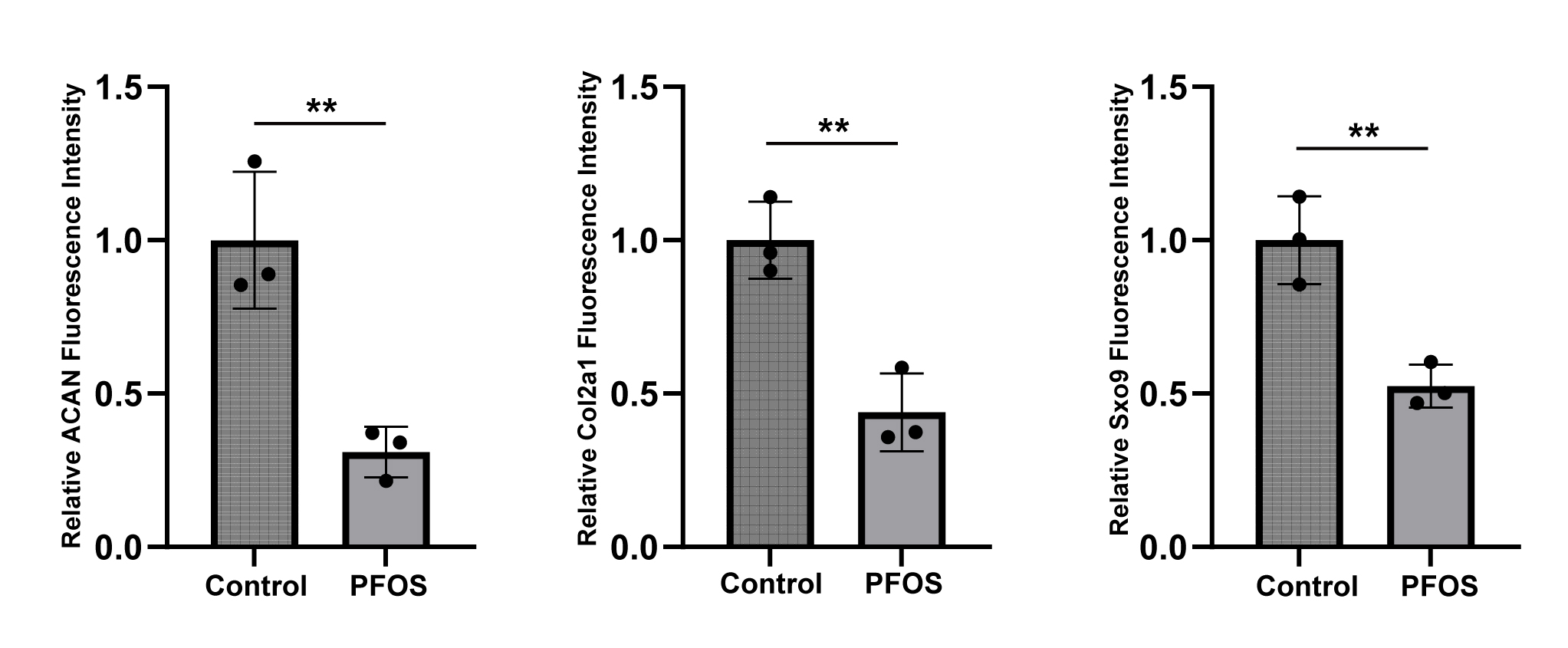

Supplement: Supplementary file 1 [file toxics-14-00575-s001.zip › Figure S1.jpg]

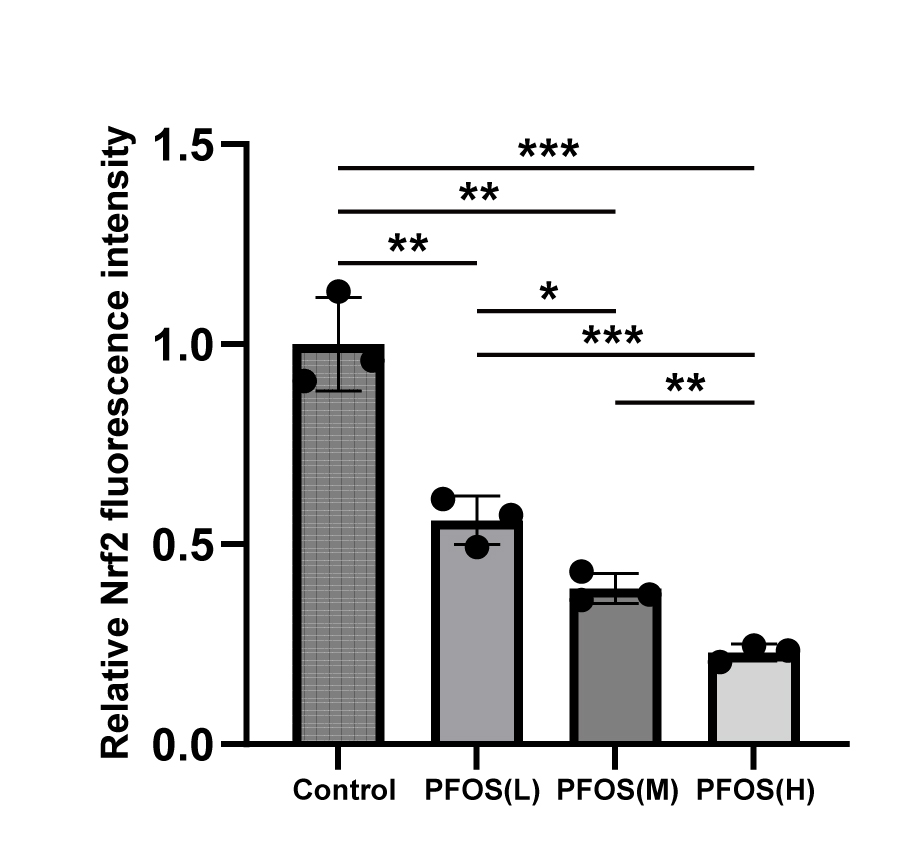

Supplement: Supplementary file 1 [file toxics-14-00575-s001.zip › Figure S2.jpg]

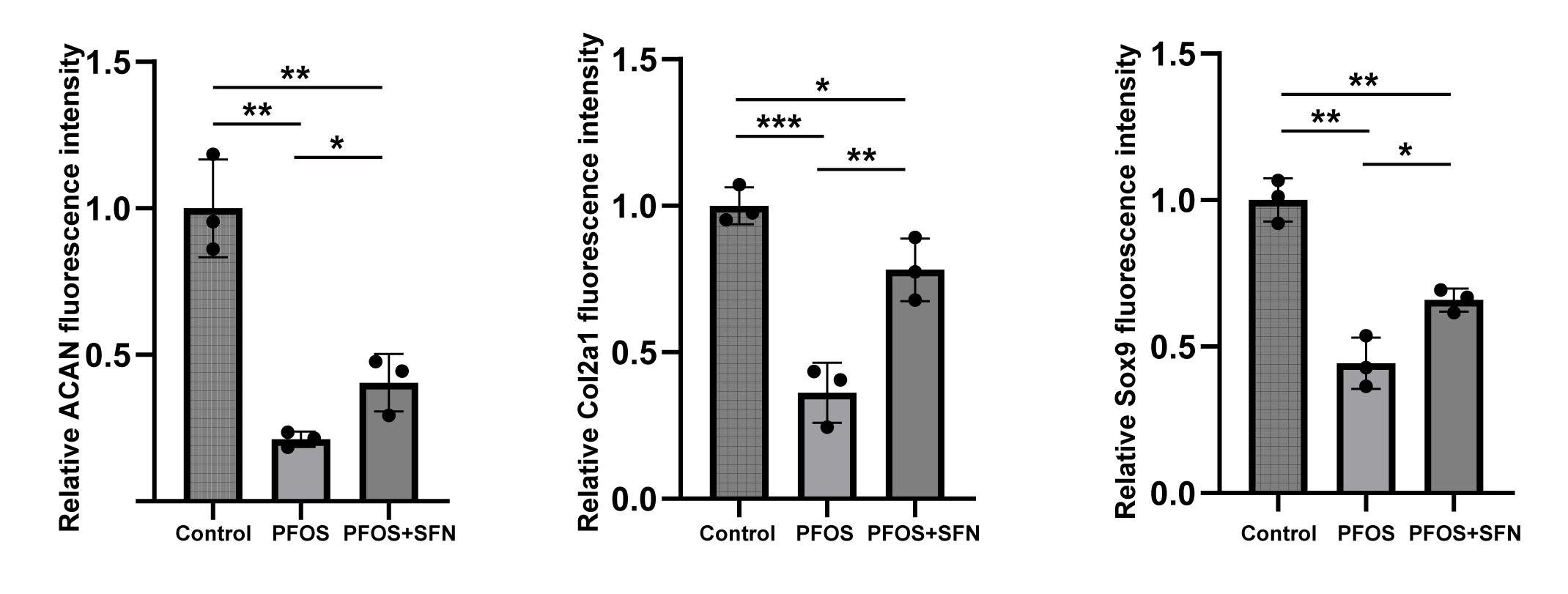

Supplement: Supplementary file 1 [file toxics-14-00575-s001.zip › Figure S3.jpg]

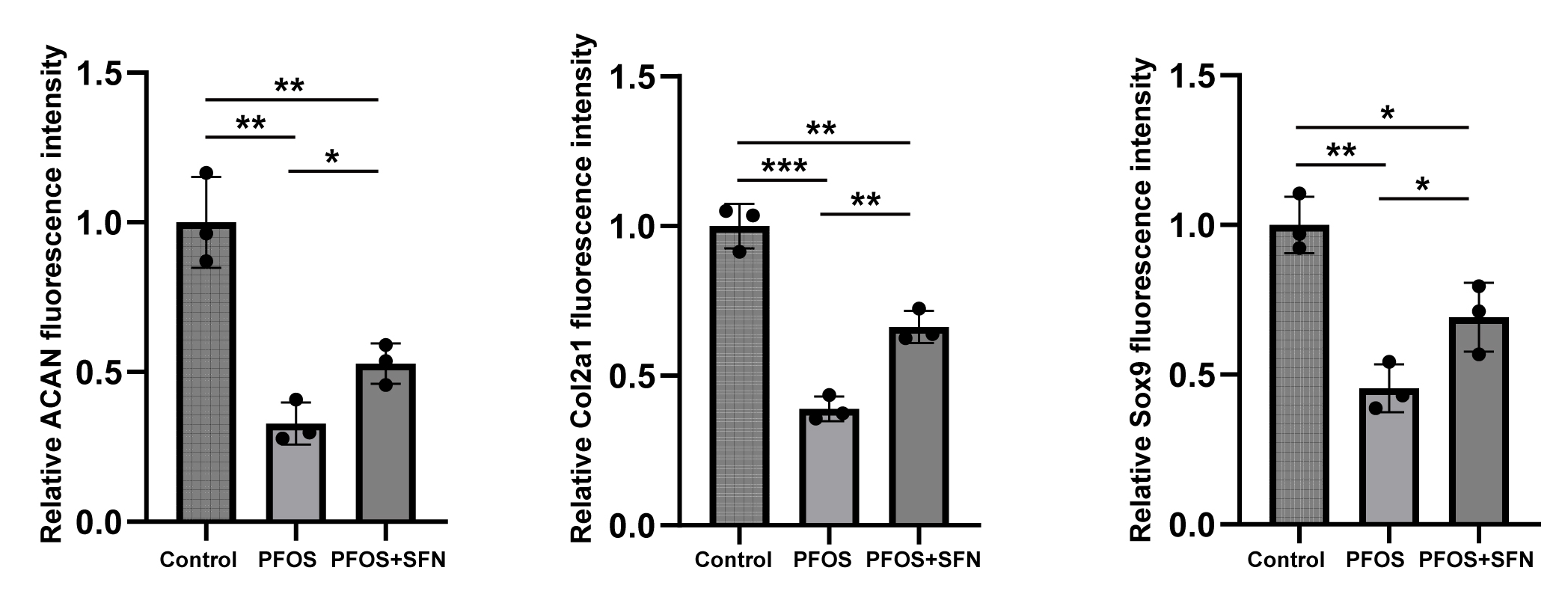

Supplement: Supplementary file 1 [file toxics-14-00575-s001.zip › Figure S4.jpg]
